# Antioxidant and anti-Alzheimer's potential of *Tetragonisca angustula* (Jataí) stingless bee pollen

**DOI:** 10.1038/s41598-023-51091-3

**Published:** 2024-01-03

**Authors:** Natalia Carine Lima dos Santos, Serena Mares Malta, Rodrigo Rodrigues Franco, Heitor Cappato Guerra Silva, Matheus Henrique Silva, Tamiris Sabrina Rodrigues, Rafael Martins de Oliveira, Thayane Nogueira Araújo, Solange Cristina Augusto, Foued Salmen Espindola, Carlos Ueira-Vieira

**Affiliations:** 1 Instituto de Biotecnologia, Universidade Federal de Uberlândia, Uberlândia, MG Brazil; 2 Instituto de Ciências Biomédicas, Universidade Federal de Uberlândia, Uberlândia, MG Brazil; 3 Departamento de Biologia, Faculdade de Filosofia, Ciências e Letras de Ribeirão Preto, Universidade de São Paulo, Ribeirão Preto, Brazil; 4 Instituto de Biologia, Universidade Federal de Uberlândia, Uberlândia, MG Brazil; 5 Laboratório de Genética, Instituto de Biotecnologia, Universidade Federal de Uberlândia, Rua Acre, Bloco 2E, Sala 226, Uberlândia, MG 38408-144 Brazil

**Keywords:** Biochemistry, Neuroscience

## Abstract

Alzheimer's disease (AD) is considered the leading cause of dementia in the elderly worldwide. It results in progressive memory loss and impairment of cognitive and motor skills, leading to a high degree of disability and dependence. The development of AD is associated with the accumulation of senile plaques in the brain, caused by the amyloidogenic pathway of the disease. Several genetic and biochemical events are linked to AD development, with oxidative stress being one of them. Due to the scarcity of drugs aimed at treating AD, antioxidant compounds are increasingly studied as therapeutic targets for the disease. In this study, we investigate the antioxidant and anti-Alzheimer potential of the *Tetragonisca angustula* (Jataí) pollen extract in *a Drosophila melanogaster* Alzheimer's model. For this purpose, we utilized a *D. melanogaster* AD-like model, which expresses genes related to the amyloidogenic pathway of Alzheimer's disease. We explored the floral origin of the collected pollen, conducted phytochemical prospecting, and evaluated its antioxidant capacity in vitro. In vivo experiments involved assessing the survival and climbing ability of the *D. melanogaster* AD-like model with various concentrations of the pollen extract. Our findings revealed that the pollen extract of *Tetragonisca angustula* exhibits a significant antioxidant response and high concentrations of important phytochemicals, such as flavonoids and polyphenols. Furthermore, it enhanced the survival rate of *D. melanogaster*, and across all concentrations tested, it improved the climbing ability of the flies after 15 days of treatment with methanolic pollen extract. Additionally, the pollen extract reduced the neurodegeneration index in histopathological analysis. Thus, our study demonstrates the potential of *Tetragonisca angustula* pollen as an important subject for further investigation, aiming to isolate molecules that could potentially serve as therapeutic targets for Alzheimer's disease.

## Introduction

Alzheimer's disease (AD) is the leading cause of dementia in the elderly, accounting for up to 70% of cases. The most significant risk factor for AD is advancing age, with exponential growth worldwide^1^. AD is characterized by progressive memory loss, impaired cognitive and motor skills, and other symptoms such as hallucinations, delusions, and depression, leading to high levels of disability and dependence^2,3^.

The pathogenesis of AD is complex and involves the aggregation of the β-amyloid peptide (Aβ), forming amyloid plaques derived from the amyloid precursor protein (APP), and the aggregation of the hyperphosphorylated tau protein, leading to the formation of intraneuronal neurofibrillary tangles. Both the β-amyloid peptide and the tau protein are considered biomarkers of the disease^4,5^. Oxidative stress is thought to play a role in the neurodegeneration of AD, as the formation of β-amyloid plaques can trigger oxidative stress events^6^. Thus, the development of therapies that can reduce oxidative stress and the production of β-amyloid peptides is of great interest^7^.

Currently, only five drugs are approved for the treatment of AD, highlighting the need for alternative therapies that can relieve symptoms but do not provide a cure or halt disease progression^8^. The human diet represents an important exogenous source of antioxidants and has drawn interest for AD therapy, with several natural compounds showing promising results in in vivo preclinical studies, alongside others already being studied in clinical trials^9^.

Bee bread is a natural supplement derived from the pollen of various plants collected by bees and mixed with their secretions. It is known to improve immune functions and has also been attributed to anti-inflammatory, antioxidant, antifungal functions, and antibacterial properties, among others^10–12^. Bee pollen contains carbohydrates, enzymes, minerals, and fatty acids, making it a natural product of high nutritional and medicinal value^13,14^. Its flavonoid, alkaloid, and phenolamine components make it an important target for biochemical study^15^.

*Drosophila melanogaster* has been employed as a model organism in research involving human neurodegenerative diseases, such as Parkinson's disease (PD), Alzheimer's disease (AD), and frontotemporal dementia (FTD), owing to its important brain complexity for the analysis of brain disorders and/or behavioral traits related to human diseases^16^. Thus, its utilization in drug and compound screening enhances the likelihood of discovering and succeeding with new drugs^17^. The presence of the Aβ peptide in the central nervous system of *D. melanogaster* can lead to the mimicry of some behavioral, pathological, and neuroanatomical changes observed in patients with Alzheimer's disease, rendering the model intriguing for evaluating responses related to treatments^18^. In this study, we administered methanolic pollen extract from *Tetragonisca angustula* Laitrelle (Apidae) to a *D. melanogaster* model of Alzheimer's disease and assessed its antioxidant properties and anti-Alzheimer’s potential.

## Results

### Palynology

**Figure 1:**
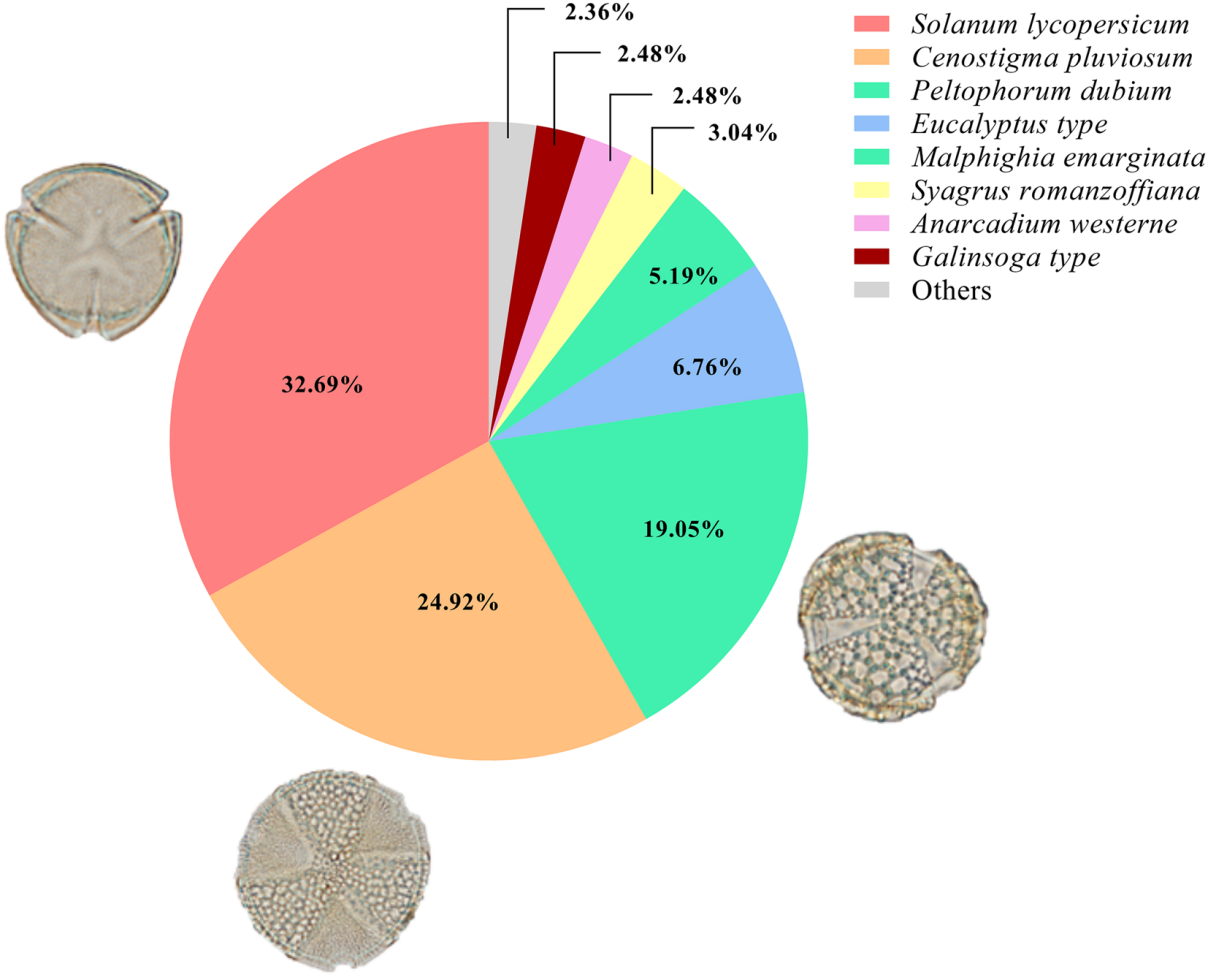
Identification and frequency percentage of pollen types found in *Tetragonisca angustula* pots.

We identified 12 different pollen types, with *Solanum lycopersicum* (Solanaceae), *Cenostigma pluviosum* (Fabaceae) and *Peltophorum dubium* (Fabaceae) being the most abundant. These three species represented 76.66% of pollen types sampled (Fig. 1).

### Phytochemical prospecting and antioxidant activity

**Figure 2:**
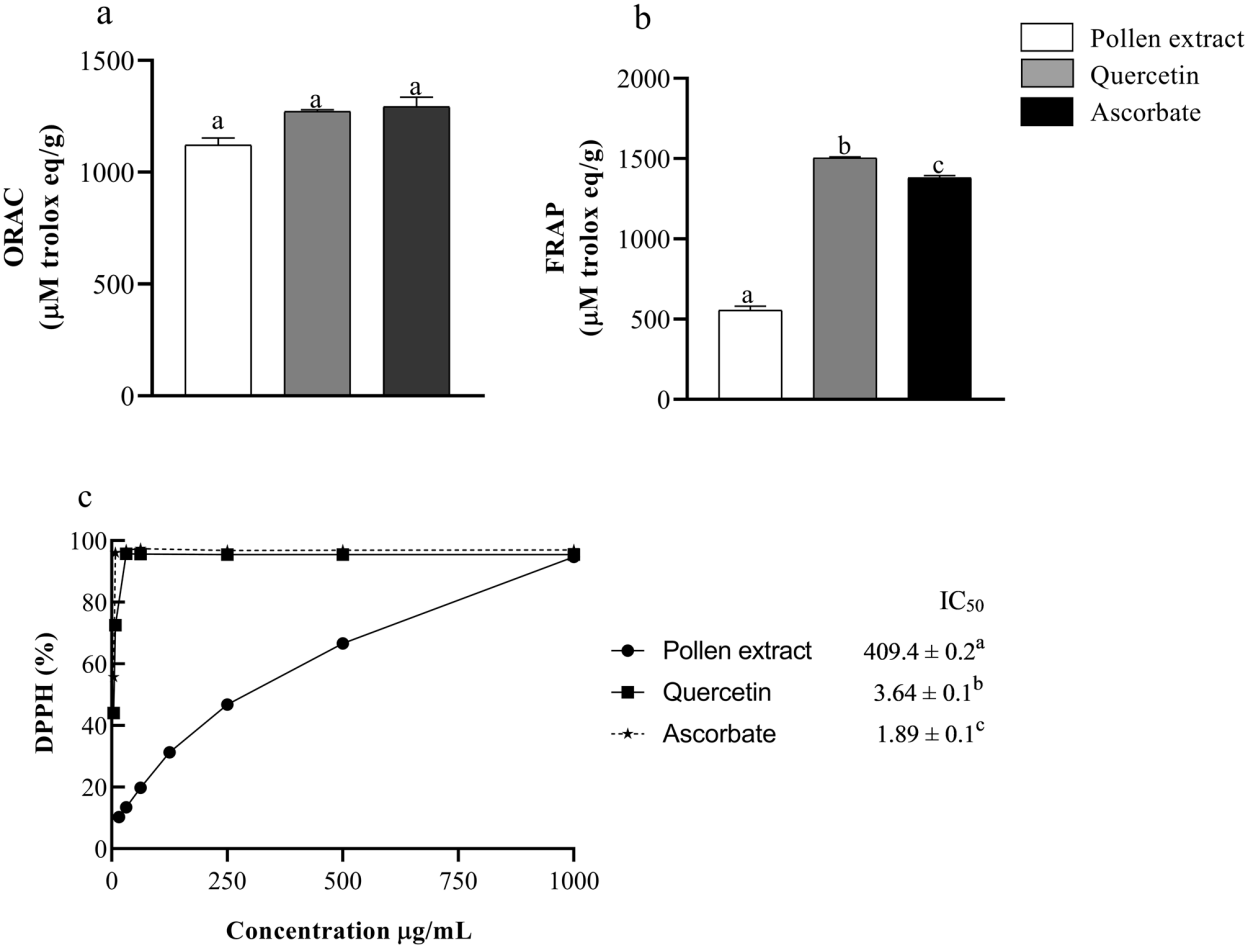
Evaluation of antioxidant capacity by methods (**A**) ORAC, (**B**) FRAP and (**C**) DPPH. The pollen extract and the flavonoid quercetin were diluted in methanol, while the ascorbate was diluted in water. Samples were diluted to 1 mg/mL for the FRAP assay and serially diluted from that concentration for the DPPH assay. For the ORAC method, samples were diluted to 100 μg/mL. Values were expressed as percent inhibition (%) for the DPPH method, with IC50 given as µg/mL, and as µmol trolox/g sample equivalents for ORAC and FRAP. Values expressed as mean ± standard error. Different letters indicate non-significant difference when compared to the control (Ascorbate) (P < 0.05).

A higher quantity of polyphenols and flavonoids was detected in the pollen derived from *Tetragonisca angustula* (Table 1). Biochemical tests were conducted to assess the antioxidant potential of the methanolic extract obtained from *T. angustula* pollen (Fig. 2). The ORAC test revealed that the pollen extract exhibited a robust capacity for antioxidant activity, akin to quercetin and ascorbate, both employed as positive controls (Fig. 2A). Assessing the antioxidant activity through iron-reducing capacity using the FRAP test (Fig. 2B), we observed the extract's effectiveness. Furthermore, when examining the antioxidant capacity via DPPH radical scavenging (Fig. 2C), the results were quantified as IC_50_ values. Notably, the minimum sample concentration required to achieve 50% activity was higher for the pollen extract compared to the values exhibited by quercetin and ascorbate.

**Table 1:** Evaluation of total polyphenols, condensed tannins, and flavonoids (mean ± standard error) content in methanolic extract of *Tetragonisca angustula.*

Polyphenols (mg GAE/g)	Condensed tannins (mg CE/g)	Flavonoids (mg QE/g)
357.5 ± 2.4	207.6 ± 5.1	277.6 ± 1.5

### Tentative of compound identification by LC–MS/MS

**Figure 3:**
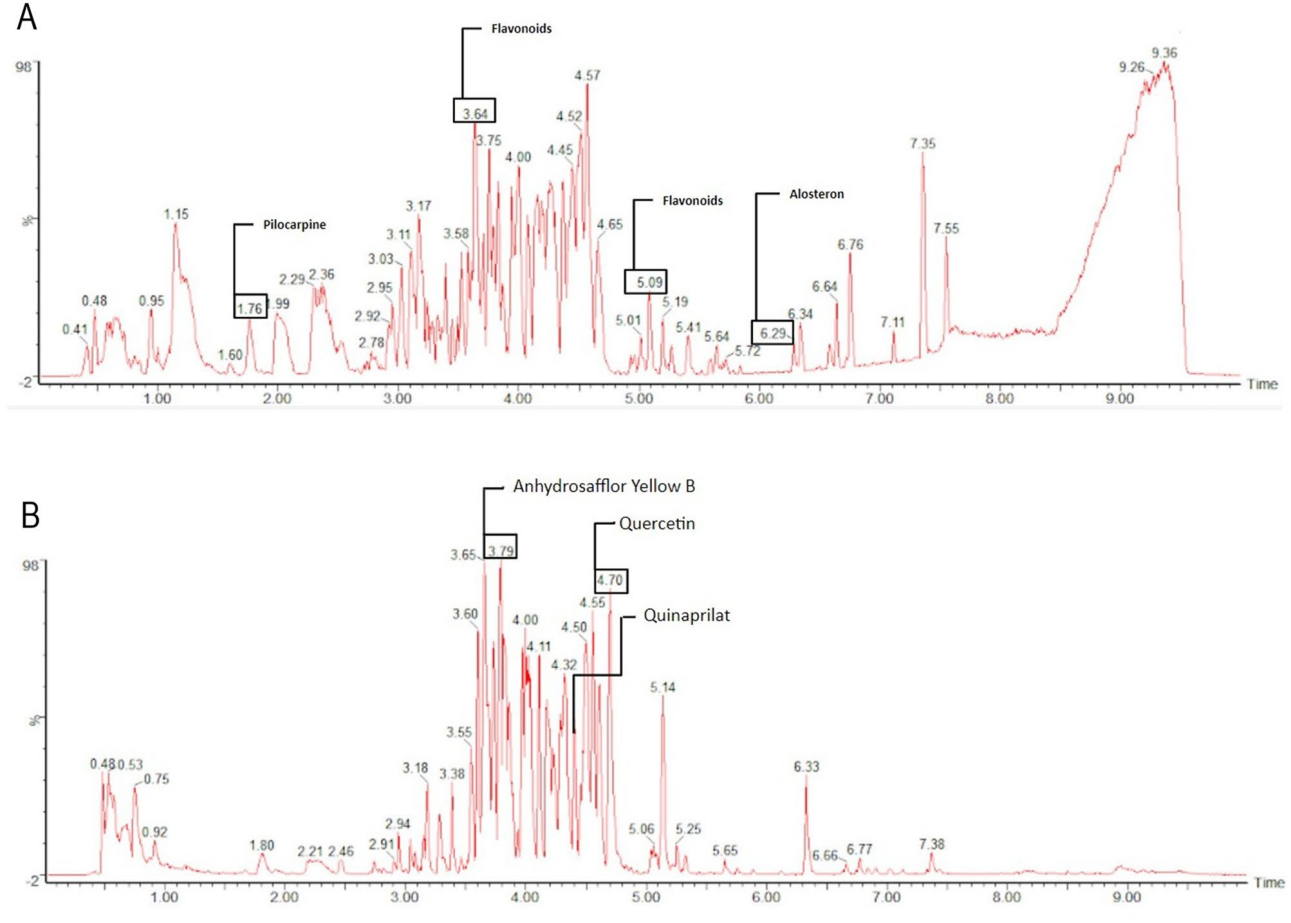
Identification of compounds by LC–MS/MS.

Our untargeted metabolomics approach unveiled peaks corresponding to flavonoids such as quercetin, alongside other compounds like alosteron, quinaprilat, anhydrosafflor yellow B, pilocarpine and others (Fig. 3).

### Methanolic pollen extract increase survival of *D. melanogaster* model of Alzheimer's (AD-like)

**Figure 4:**
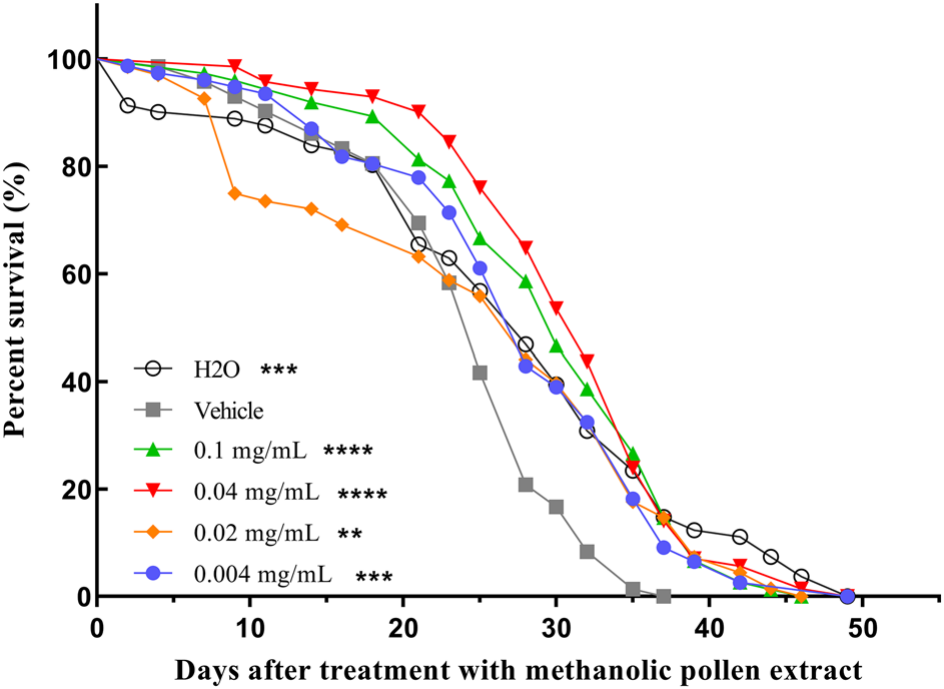
Survival rate of AD-like flies treated with methanolic pollen extract at concentrations 0.1 mg/mL, 0.04 mg/mL, 0.02 mg/mL and 0.004 mg/mL, vehicle and untreated groups (N = 90 for each group). Statistical significance: (**) P < 0.01, (***) P < 0.001 and (****) P < 0.0001 (log-rank Mantel–Cox test).

**Figure 5:**
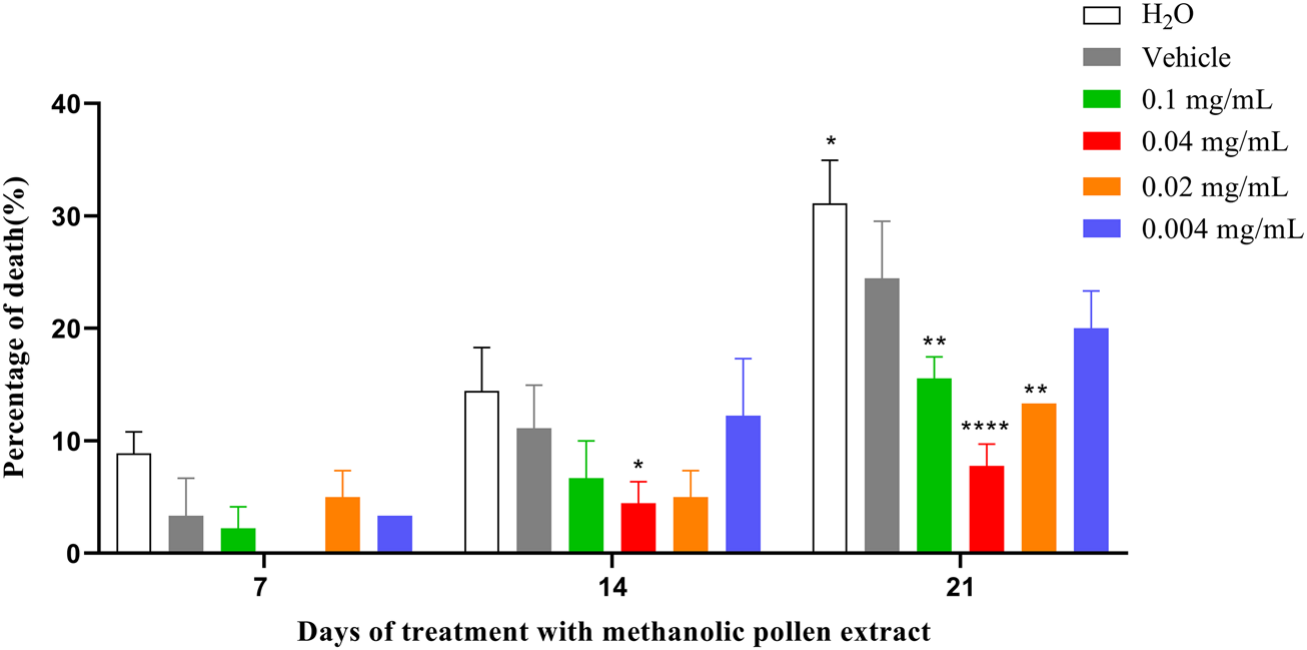
Percentage of death of AD-like flies treated with methanolic pollen extract at concentrations of 0.1 mg/mL, 0.04 mg/mL, 0.02 mg/mL and 0.004 mg/mL, vehicle and untreated (N = 90 for each group) in time interval of 7, 14 and 21 days. Statistical significance: P < 0.05 (*), (**) P < 0.01 and P < 0.0001 (****).

We assessed the impact of *T. angustula* pollen methanolic extract on fly survival. Our findings demonstrated a significant distinction between untreated flies (control) and those solely treated with the vehicle (Fig. 4, P < 0.001). Notably, a significant decline in survival among these flies was observed starting on the 20th day of vehicle treatment in AD-like flies. Additionally, we examined the mortality rate at intervals of 7, 14, and 21 days for each treatment. Results revealed that in the methanolic pollen extract treatments of 0.1 mg/mL (P < 0.01), 0.04 mg/mL (P < 0.0001), and 0.02 mg/mL (P < 0.01), the percentage of deaths was statistically lower after 21 days compared to other treatments and the untreated group (Fig. 5).

### AD-like model validation

**Figure 6:**
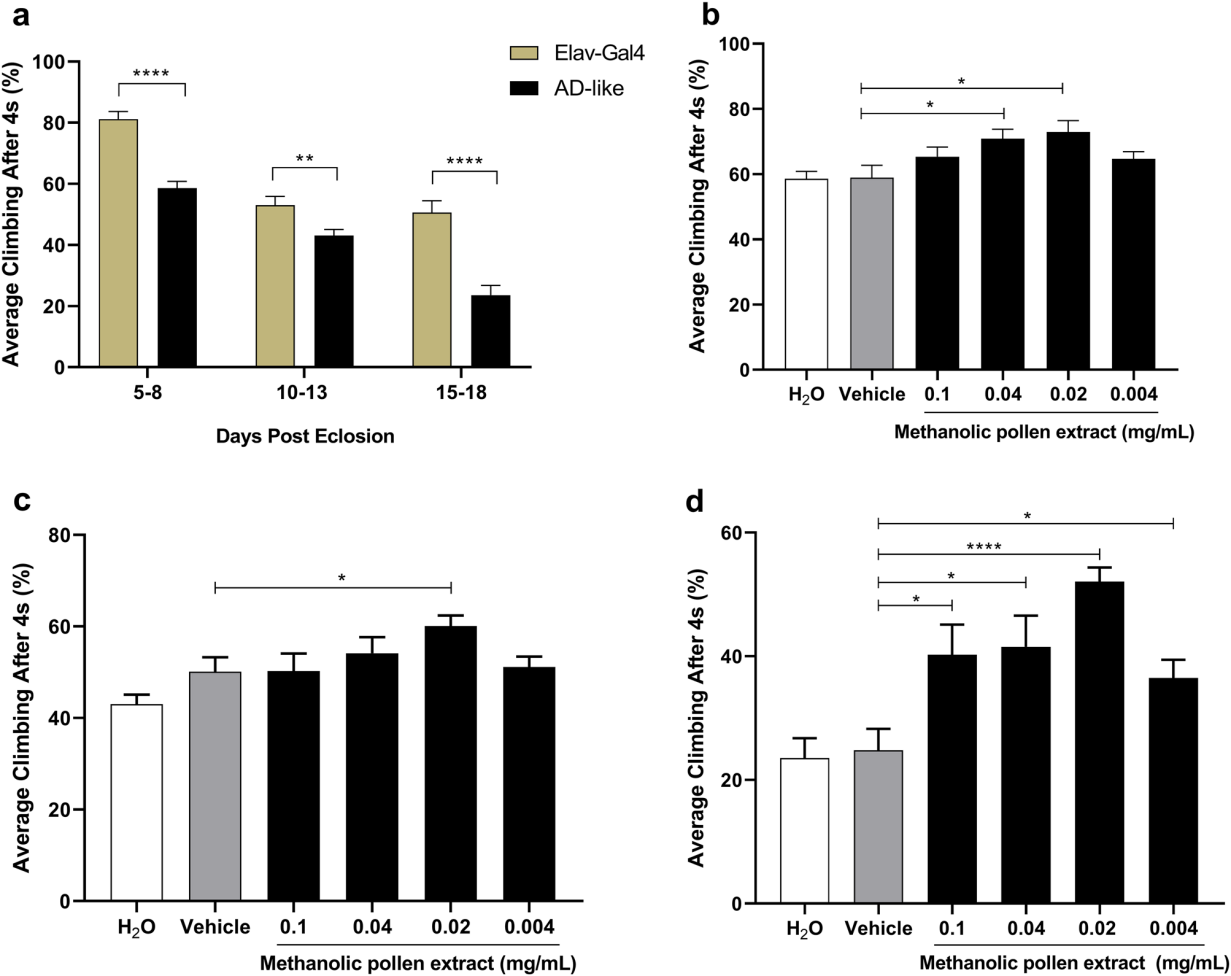
AD-like model validation and climbing ability of AD-like flies after treatment with methanolic pollen extract. (**A**) Comparison of climbing ability between Elav-Gal4 and AD-like genotypes at 5–8, 10–13 and 15–18 days post eclosion. (**B**) 5 days of treatment with 0.1 mg/mL, 0.04 mg/mL, 0.02 mg/mL and 0.004 mg/mL. (**C**) 10 days of treatment (**D**) 15 days of treatment. Data are presented as mean ± SEM and significance values are represented as P < 0.05 (*), P < 0.01 (**) and P < 0.0001 (****) Unpaired two-tailed t-test.

The Rapid Iterative Negative Geotaxis (RING) test was conducted to evaluate the motor ability of AD-like flies compared to the elav-Gal4 control genotype at various time intervals. The AD-like genotype exhibited a significant decline in climbing ability at 5–8, 10–13, and 15–18 days post-eclosion (d.p.e) when compared to elav-Gal4 (Fig. 6A). Thus, confirming the validation of the AD-like model.

### Methanolic extract of pollen promotes improvement in behavioral test and histopathological analysis to measure neurodegeneration in AD-like flies

**Figure 7:**
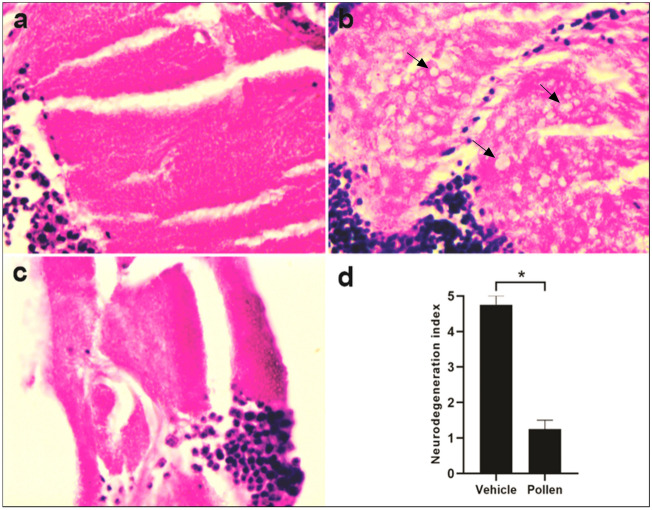
Histology of the brain of AD-like model fly treated with methanolic pollen extract. Representative 3-μm paraffin sections brain of flies 15-days after hatching. Illustrative images of histopathologic analysis of (**a**) elav-Gal4, magnification of ×100; (**b**) AD-like treated with vehicle (tween), ×100—arrows indicate vacuolar lesions, and (**c**) AD-like flies treated with pollen, ×100. (**d**) Neurodegeneration index of AD-like flies treated with vehicle (tween) and pollen based on histopathology analysis, according to vacuolar lesions. 0 indicates no lesions, and 5 indicates neurodegenerative phenotype (n = 3, at 15 days after treatment). Data are shown as the mean ± SEM. The statistical significance is indicated as * for P < 0.05 (Mann Whitney test).

After five days of methanolic pollen extract treatment, AD-like flies displayed a significant increase in climbing ability at concentrations of 0.04 mg/mL and 0.02 mg/mL (P < 0.05) compared to the vehicle (Fig. 6B). Results obtained from the climbing test after ten days of methanolic pollen extract treatment (Fig. 6C) indicated that only the 0.02 mg/mL concentration showed enhanced climbing ability compared to the vehicle (P < 0.05). By the fifteenth day of treatment, it became evident that all tested concentrations had improved the climbing ability of AD-like flies (Fig. 6D, P < 0.05). Furthermore, the treatment with methanolic pollen extract on the AD-like model at fifteen days post-eclosion exhibited improvements in damaged tissue and a decrease in the neurodegenerative index (Fig. 7).

## Discussion

In this study, we examined the diversity of pollen, the concentration of polyphenols, and the impact of methanolic pollen extract on an Alzheimer's-like model. Our findings unveiled the prevalence of three pollen types, a high polyphenol and flavonoid content in the bee bread of *T. angustula*. The methanolic extract exhibited antioxidant potential and proved effective in enhancing climbing ability and ameliorating damaged brain tissue in *Drosophila melanogaster*, an alternative model of Alzheimer's disease.

Stingless bees play a crucial role as efficient pollinators, vital for biodiversity and the preservation of both native and exotic plant species within each region. *Tetragonisca angustula* (popularly known as Jataí) stands out among stingless bee species in Brazil due to its broad dietary habits, significantly contributing to the pollination of tropical flora^19^. Through palynological analysis, we identified several pollen types, although their diversity was lower compared to studies conducted in wild environments^20–23^.

The most prevalent pollen type found in the sample was *Solanum lycopersicum* (tomato). This could potentially be sourced from an urban agriculture area located within 200 m of the urban meliponary where the samples were collected. Despite *T. angustula* not engaging in buzz pollination, this species might visit *S. lycopersicum* to gather residual pollen left in the flowers^24^. The other predominant pollen types were *Cenostigma pluviosum* and *Peltophorum dubium*, native plants commonly utilized in urban landscaping. This underscores the significance of urban agriculture and gardens as a food source for nurturing bees in urban areas^25^.

We also assessed the antioxidant potential of bee pollen through FRAP, ORAC, and DPPH biochemical assays. Our results exhibited considerable antioxidant potential in the methanolic pollen extract. Utilizing the ORAC test, we discovered that *T. angustula* pollen demonstrates antioxidant activity comparable to quercetin (a positive control used in the test, also detected in the pollen extract through LC/MS/MS analysis) and ascorbate. This is a compelling biochemical finding considering that the tests were conducted with the crude extract. However, in the FRAP and DPPH tests, the results indicated a lower performance of the pollen extract compared to the positive controls, possibly due to the utilization of the crude extract, as these tests yield better outcomes when isolated compounds are used. Other studies have demonstrated via these tests that pollen exhibits outstanding in vitro antioxidant activity, potentially surpassing that of certain fruits or fruit parts reported in the literature for their high antioxidant capacity^26–30^.

Based on our in vitro findings, we observed that pollen collected from *T. angustula* harbors a high concentration of polyphenols and flavonoids, secondary metabolites prevalent in human diet and nutrition^31,32^. This profile aligns with other studies involving stingless bees (*Mellipona scutellaris*, *Cephalotrigona capitata*, and *Apis mellifera*) analyzing honey properties, which also identified several flavonoids such as quercetin, kaempferol, and hesperidin, known not only for their antioxidant properties but also as anticancer, anti-inflammatory, and antimicrobial agents^33^. Our study revealed significant concentrations of polyphenols and flavonoids in pollen compared to another study using propolis extract from *T. angustula*^34^. Our results confirm that pollen from this stingless bee could serve as a valuable source of natural antioxidant compounds.

Gallic acid, ferulic acid, and p-coumaric acid are also reported to exhibit antioxidant properties in the honey of Africanized honeybee (*Apis mellifera*) from Brazil, underscoring the floral origin's role in the characteristics of bee-derived food products^35^. Tomato (*S. lycopersicum*), the predominant pollen in our sample, is recognized for its substantial content of polyphenols, particularly flavonoids in its fruit^36^, and pollen^37^. While there is limited data on metabolites or antioxidant effects in pollen from *C. pluviosum* and *P. dubium*, it is known that flavonoids play a crucial role in regulating pollen tube growth and integrity^38^. Consequently, it is expected that pollen contains a notable amount of flavonoids. Moreover, fermentation facilitated by microorganisms can heighten the concentration of these compounds present in pollen^39^.

The association between microorganisms and hives suggests mutualistic interactions between involved organisms, potentially benefiting the nutritional composition of bee pollen, along with environmental floral traits. In terms of biochemical properties, phenolic compounds constitute a well-studied group, particularly recognized for their antioxidant potential^40^, with numerous studies showcasing their abundance in bee pollen collected from diverse locations^41^.

Unlike honeybees, stingless bees store pollen in cerumen pots, where it undergoes fermentation due to microorganism presence^42^. The biochemical traits of pollen vary depending on the local flora of collection, potentially influenced by fermentative microorganism action. This fermentation process elevates the nutritional value of pollen, and various bacterial species are reported in stingless beehives, including fermentative, lactic acid, and non-lactic acid bacteria. Previous research has highlighted bacteria such as *Lactobacillus musae*, *Lactobacillus crustorium*, *Lactobacillus mindesis*, *Leuconostosc mesenteroides*, *Enterococcus faecalis*, *Fructobacillus fructosus*, *Bacillus megaterium*, *Bacillus punilus*, *Bacillus flexus*, *Bacillus coagulans*, *Bacillus safensis*, *Bacillus amyloliquefaciens*, and *Starmerella meliponinorum*, the latter specifically isolated from *Tetragonisca angustula* in Brazil^14,43–47^.

In Brazil, bee pollen has gained popularity as a potential remedy for neurodegenerative conditions. However, there's a lack of published research on its effects specifically concerning diseases affecting cognitive functions. A recent study highlighted the potential of commercial bee pollen extract from honeybees to alleviate impairment and enhance cognitive functions in long-term rat models. This was identified as a significant therapeutic agent for treating mild cognitive impairment or conditions arising from cholinergic dysfunction^48^. In our study, we demonstrated that the methanolic extract of pollen from *T. angustula*, a stingless bee species, enhanced climbing ability and ameliorated damaged tissue in the *D. melanogaster* AD-like model, a primary behavioral assessment for screening novel anti-Alzheimer compounds in flies^49^.

Flavonoids, among the bioactive compounds found in bee pollen, have previously shown importance in research on cognitive decline. Preclinical studies investigating Alzheimer's disease models with increased β-amyloid production suggest that flavonoids could potentially delay the onset or slow the progression of the disease^50,51^. We identified polyphenols, condensed tannins, and flavonoids in the methanolic extract of *T. angustula* pollen using biochemical assays. LC–MS/MS analysis identified specific compounds within these groups. It's noteworthy that since the stingless bee *T. angustula* collects and stores pollen in cerumen pots for fermentation, the compounds detected in the metabolomics analysis may originate from pollen or from the metabolic activity of microorganisms.

Our results underscore the efficacy of the methanolic extract of *T. angustula* pollen in enhancing climbing ability and repairing damaged brain tissue in the *Drosophila melanogaster* Alzheimer's disease model. This highlights the biotechnological potential inherent in Brazil's biodiversity and in products harvested from indigenous bee colonies. Our study exhibits promise for further assessment in murine models and biochemical assays aimed at identifying and isolating bioactive compounds. It's important to note that additional investigations are necessary to determine the exact mechanism through which bee pollen improves cognitive function in neurodegenerative diseases. Nonetheless, our study offers crucial insights into the potential utilization of bee pollen as a therapeutic tool for addressing cognitive impairment in such conditions.

## Material and methods

### Drugs and reagents

Analytical-grade reagents and solvents were purchased from Sigma (Sigma, St Louis, MO, USA) or local suppliers. Milli-Q^®^ water, obtained by deionized and filtered water on a Millipore filter, was used throughout the study.

### Extract preparation

The pollen was harvested from two colonies of *Tetragonisca angustula* stingless bees located in an urban meliponary in Uberlândia city, Minas Gerais State, Brazil, in June 2020. The collected pollen, obtained from sealed pots, totaled 83 g and was transferred to a beaker. Subsequently, 581 mL of absolute methanol (in a ratio of 1:7) were added to the beaker containing the pollen and stirred for 30 min. Afterward, the mixture was kept in the absence of light for 88 h at room temperature. The resulting liquid was then filtered and processed using a rotary evaporator. The obtained extract underwent lyophilization and freezing, resulting in the production of 11 g of crude extract.

### Pollen analysis

The crude pollen samples underwent acetolysis and were then mounted on microscopic slides, following the method described by Louveax et al.^52^. The identification of pollen types was based on the literature^53^, the slide collection (Funed-Pol) available on the species network (http://www.splink.org.br), reference specimens housed at the Laboratory of Morphology, Microscopy, and Plant Imaging (LAMOVI) at the Federal University of Uberlandia, and observation of plants around the beehive. For the quantitative pollen analysis, two slides were prepared from the sample. Each glass slide was divided into four quadrants, and 100 grains per quadrant were counted, totaling 800 pollen grains.

### Phytochemical prospecting

To determine the total concentrations of polyphenols, condensed tannins and flavonoids in the extract, we used the method by Zou et al.^54^ modified by Franco et al.^55^ in 96-well microplates. The extract was diluted in methanol at a concentration of 10 mg/mL. All analyzes were performed in triplicate.

### Determination of the concentration of polyphenols

To determine the total phenols, we utilized the Folin–Ciocalteu reagent, involving the reduction of Folin's reagent (a mixture of phosphomolybdic and phosphotungstic acids) by phenolic compounds. The procedure commenced by adding 5 µL of the sample, 25 µL of an aqueous Folin–Ciocalteu reagent solution, and 195 µL of milli-Q water into each well. The plate was then incubated for 6 min at 25 °C. Following incubation, 75 μL of sodium carbonate (7%) was introduced, and the plate underwent another 2-h incubation at 25 °C in the absence of light.

Measurements were taken by assessing the absorbance using a spectrophotometer at a wavelength of 760 nm. A parallel procedure was conducted using 5 µL of methanol to derive the blank. Total phenol content was determined using an analytical curve constructed with gallic acid standards, spanning concentrations of 31.25; 62.5; 125; 250; 500; 1000; and 2000 µg/mL. The findings were expressed as milligrams of gallic acid equivalents per gram of the sample (mg GAE/g).

### Determination of the concentration of condensed tannins

Each well received 10 µL of the sample, 200 µL of a 4% methanolic vanillin solution, and 100 µL of concentrated HCl. The plate was incubated for 15 min at 25 °C, and the absorbance was measured at a wavelength of 500 nm using a spectrophotometer. The same process was repeated with 10 µL of methanol to establish the blank. Total phenol content was quantified using an analytical curve constructed with catechin standards (CE), ranging from concentrations of 31.25; 62.5; 125; 250; 500; 1000; to 2000 µg/mL. The results were expressed as milligrams of catechin equivalents per gram of the sample (mg CE/g).

### Determination of the concentration of flavonoids

To determine the concentration of flavonoids, 30 µL of the sample, 180 µL of milli-Q water, and 10 µL of 5% sodium nitrite solution were added to each well. The plate was then incubated for 6 min at 25 °C in the absence of light. Subsequently, 20 μL of 10% aluminum chloride solution was added, and the plate was incubated for an additional 6 min at 25 °C in the dark. Absorbance was measured at a wavelength of 425 nm using a spectrophotometer. The same procedure was repeated using 30 µL of methanol to establish the blank. Flavonoid content was determined using an analytical curve constructed with a standard quercetin at concentrations of 31.25, 62.5, 125, 250, 500, 1000, and 2000 µg/mL. The results were expressed as milligrams of quercetin equivalents per gram of the sample (mg QE/g).

### Antioxidant assays

For the antioxidant assays, the pollen extract and the flavonoid quercetin were dissolved in methanol, while ascorbate was dissolved in water, all at a concentration of 1 mg/mL for the FRAP (Ferric Reducing Antioxidant Power) method and 100 µg/mL for the ORAC (Oxygen Radical Absorbance Capacity) method. For the DPPH (2,2-diphenyl-1-picrylhydrazyl) free radical scavenging method, samples were serially diluted starting from a concentration of 1 mg/mL to determine the IC_50_ value. All analyses were conducted in triplicate. The results for the DPPH assay were expressed as percentage (%) antioxidant capacity, with IC50 reported in µg/mL, while the results for the FRAP and ORAC assays were presented as trolox equivalents (µmol trolox/g).

### Iron reduction capacity (FRAP)

Analysis of antioxidant capacity by the FRAP method was based on Benzie and Strain^56^. Initially, 250 μL of FRAP reagent consisting of 10 volumes of sodium acetate buffer (0.3 mol/L and pH 3.6), 1 volume of 10 mmol/L of TPTZ (2,4,6-tri (2 piridil)-s-triazina) solution and 1 volume of aqueous ferric chloride solution (20 mmol/L)], 10 μL of extract/partition and 25 μL of milli-Q water was incubated for 6 min at 37 °C. The absorbances were measured in a spectrophotometer at 593 nm and the antioxidant capacity was determined using an analytical curve constructed with trolox. Sodium acetate buffer was used as a blank.

### Oxygen radical absorbance capacity (ORAC)

The assessment of antioxidant capacity by the ORAC method was based on Prior et al.^57^. In this assay, all reagents were prepared in phosphate buffer at 75 mmol/L and pH 7.4. Initially, 25 μL of extract/partition were mixed with 150 μL of fluorescein (0.085 nmo/L) and incubated at room temperature for 15 min. Then, 30 μL of the 153 mmol/L Azobis solution (2,2′-Azobis(2-methylpropionamidine) dihydrochloride) was added to start the reaction. The fluorescence intensity (485 nmex/528 nmem) was measured in a spectrofluorimeter and checked every 1 min and 30 s for 90 min. The blank was performed replacing the extract/partition with phosphate buffer. The loss of fluorescein fluorescence was measured using the area under the curve calculation and the antioxidant capacity was determined using an analytical curve constructed with trolox.

### Sequestration of DPPH

The DPPH method was based on the technique described by Sharma and Bhat^58^ and adapted by Franco et al.^59^. Samples were incubated with a methanolic solution of 60 mM DPPH at 30 °C for 20 min in the absence of light. The assay started with 250 μL of extract/partition solubilized in 750 μL of methanolic DPPH solution. The mixture containing extract/partition and ascorbic acid was incubated at 30 °C, in the absence of light, for 20 min. The reduction in the absorbance of the mixture was measured in a spectrophotometer at 517 nm.

### Liquid chromatography with tandem mass spectrometry LC–MS/MS

The lyophilized methanolic extract was reconstituted in methanol at a concentration of 10 mg/mL, and 10 µL of the sample volume was loaded onto the UPLC-ESI-QTOF-MS system (Waters). Prior to injection, the columns were primed with 100% mobile phase A (a mixture of 97:3 water with 0.1% formic acid and acetonitrile) and 0% mobile phase B (acetonitrile containing 0.1% formic acid). The column temperature was set to 45 °C. Metabolites were separated on an analytical column ACQUITY UPLC H-Class HSS T3 C18 column, with a particle size of 1.8 µm and dimensions of 2.1 × 100 mm (Waters, Corp., Milford, USA), using a flow rate of 0.5 mL/min and a gradient elution of 100% A from 0.0 to 1.0 min, 5% A and 9% B from 1.0 to 8.0 min, maintaining 5% A and 9% B from 8.0 to 8.5 min, returning to 100% A from 8.5 to 8.6 min, and holding at 100% A from 8.6 to 10.0 min. Data-independent acquisition mode (MSE) was employed by operating the instrument in positive and negative ion V modes, using MS and MS/MS functions in 0.2 s intervals with low-energy set at 0 V and high-energy collision set between 20 and 40 V to capture the mass-to-charge ratio (m/z) of the metabolites. The capillary voltage was set at 40 V, and the source temperature was maintained at 100 °C. To correct for mass drift, Leucine Enkephalin (556.2771 Da) was infused as an internal mass calibrant at a rate of 10 µL/min through the Lockspray ion source every 30 s. Ion signal data were collected between 50 and 1200 m/z values, and three LC–MS/MS runs were performed in each mode.

### Tentative of compound identification by untargeted LC–MS/MS data analysis

The raw data were processed and analyzed in Progenesis QI 3.0.3. (Nolinear Dynamics, Waters). To tentative identify compounds, we compared ours data with all LC–MS/MS Positive Mode and LC–MS/MS Negative Mode from three different databases: MoNA MassBank of North America (https://mona.fiehnlab.ucdavis.edu/downloads), Biomolecules from Waters and the Natural Products (https://marketplace.waters.com/apps/159222/natural-products-applicationsolution-media#!overview). The search parameters were set as follow: the mass error was 5 ppm, isotope similarity above 95 and adducts only M+H or M−H. The duplicates information from different databases were removed.

### *D. melanogaster* stocks

The stock of flies was obtained from the Bloomington Stock Center: w^1118^ (BL #3605), UAS-BACE-1, UAS-APP (#33797) and elav-GAL4 (#5146). The flies used were maintained in standard cornmeal medium (soybean powder 0.01%, glucose 7.2%, agar 0.6%, cornmeal 0.073%, yeast 0.018%, nipagin 0.06% and acid solution 0.05% m/v) and kept in an incubator with a light/dark cycle 12 h:12 h at 25 °C.

### AD-like model

To create AD-like flies, we conducted crosses utilizing virgin females from the elav-Gal4 lineage and males from the UAS-BACE-1; UAS-APP lineage. In the F1 generation, selection occurred during the pupal stage based on phenotype, discarding tubby-type pupae and choosing elongated ones (wild phenotype) as the AD model (elav-Gal4/+; UAS-BACE-1, UAS-APP/+). Control flies were generated through the cross of Elav-Gal4 with *w*^*1118*^ (elav-Gal4/+genotype).

### Treatments with methanolic pollen extract from *T. angustula*

For the in vivo assay, we utilized four concentrations of the extract: 0.1 mg/mL, 0.04 mg/mL, 0.02 mg/mL, and 0.004 mg/mL. These concentrations were diluted using 0.01% Tween as the vehicle. Each concentration was added to 5 g of enriched puree medium, consisting of 75% instant mashed potato, 15% yeast extract, 9.3% glucose, and 0.07% nipagin. The treatment was administered to the flies from 0 to 3 days post-eclosion, and the medium was refreshed every 2 days. Alongside the groups of flies tested for each concentration (N = 90), we also included groups subjected to water (control) and 0.01% Tween (vehicle) for comparison.

### Survival assay

To assess the survival rate, we employed 90 male and female AD-like flies, each subjected to the treatments described earlier. The treatments were alternated, and we recorded the number of deceased flies every 2 days until the entire population expired.

### Rapid iterative negative geotaxis (RING) assay

For the climbing tests, we exclusively used male AD-like and control flies, conducting the test in triplicate, with 30 flies in each vial. The flies were positioned on a suitable support capable of accommodating 12 vials. During the pre-test (acclimatization phase), the flies were exposed to light in a quiet environment for 20 min. Subsequently, the support containing the vials was tapped against the bench three times, after which the climbing activity of the flies up to 5 cm in four seconds was observed. This process was repeated and recorded five times, with a 1-min interval between each repetition. We evaluated the climbing ability of the flies at 5, 10, and 15 days post-treatment. Video analysis was conducted using QuickTime Player 7.7.9 software.

### Histological analysis

For histological analysis, five 15-day-old adult flies from each control/treatment group, belonging to the elav-GAL4;UAS-BACE1, UAS-APP strain, were collected, anesthetized using ethyl ether, and fixed in Carnoy solution (6:3:1, 99% ethanol, chloroform, and glacial acetic acid) for 24 h. Subsequently, they were processed through a series of ethyl alcohol concentrations (70%—2×, 80%—2×, 90%—2×, absolute—2×), xylene (2×) for 15 min per repetition, and 60% liquid paraffin (2×) for 30 min each. The flies' heads were embedded in paraffin, and 3 μm thick sections were obtained using a semi-automatic microtome (SLEE CUT5062). These sections were then hydrated, stained with hematoxylin and eosin, mounted, and captured using a light photomicroscope. Neuropile images from three or more adult flies were used to determine the neurodegenerative index, categorized as normal, low, moderate, or severe based on vacuolar lesions^60^.

### Statistical analysis

For the phytochemical prospection test and antioxidant activity assays, statistical analyses and graphics were performed using GraphPad Prism 8.0 software. Data were expressed as mean ± standard error of the mean (SEM). In the antioxidant activity assays, the results were submitted to one-way ANOVA with Tukey's test for multiple comparisons between sample means. P values < 0.05 were considered significant. For the other experiments, it was evaluated whether the results had a normal distribution (parametric or non-parametric) using the D'Agostino and Pearson test.

### Supplementary Information

Supplementary Information.

## Data Availability

All data generated or analyzed during this study are included in this published article.
