# Supplementary material for: Antioxidant and anti-Alzheimer's potential of Tetragonisca angustula (Jataí) stingless bee pollen
Source: Sci Rep. 2024 Jan 3;14:308. doi: 10.1038/s41598-023-51091-3 (PMC10764861; doi:10.1038/s41598-023-51091-3)
Supplement: Supplementary file 1 — Supplementary Information. [file 41598_2023_51091_MOESM1_ESM.pdf]

Antioxidant and anti-Alzheimer's potential of *Tetragonisca angustula* (Jataí) stingless bee pollen

Natalia Carine Lima dos Santos[2*], Serena Mares Malta[1], Rodrigo Rodrigues Franco[1], Heitor Cappato Guerra Silva[1], Matheus Henrique Silva[1], Tamiris Sabrina Rodrigues[1], Rafael Martins de Oliveira[2], Thayane Nogueira Araújo[3], Solange Cristina Augusto[4], Foued Salmen Espindola[1] & Carlos Ueira-Vieira[1*].

[1]Instituto de Biotecnologia, Universidade Federal de Uberlândia, Uberlândia, MG, Brasil.

[2]Instituto de Ciências Biomédicas, Universidade Federal de Uberlândia, Uberlândia, MG, Brasil.

[3]Departamento de Biologia, Faculdade de Filosofia, Ciências e Letras de Ribeirão Preto, Universidade de São Paulo, Ribeirão Preto, Brasil.

[4]Instituto de Biologia, Universidade Federal de Uberlândia, Uberlândia, MG, Brasil.

*Correspondence to: Natalia Carine Lima dos Santos (carinenatalia@yahoo.com.br) and Carlos Ueira-Vieira (ueira@ufu.br).

Laboratório de Genética, Instituto de Biotecnologia, Universidade Federal de Uberlândia, Rua Acre, Bloco 2E, sala 226, Uberlândia, MG, Brasil, 38408-144.

Table 1. Evaluation of total polyphenols, condensed tannins, and flavonoids (mean ± standard error) content in methanolic extract of *Tetragonisca angustula*.

**Polyphenols (mg GAE/g)**	**Condensed tannins (mg CE/g)**	**Flavonoids (mg QE/g)**
357.5±2.4	207.6±5.1	277.6±1.5
